# Nanoimpact in Plants: Lessons from the Transcriptome

**DOI:** 10.3390/plants10040751

**Published:** 2021-04-12

**Authors:** Susana García-Sánchez, Michal Gala, Gabriel Žoldák

**Affiliations:** 1 Center for Interdisciplinary Biosciences, Technology, and Innovation Park P.J. Šafárik University, Trieda SNP 1, 040 11 Košice, Slovakia; 2 Department of Biophysics, Faculty of Science, P. J. Šafárik University, Jesenna 5, 040 01 Košice, Slovakia; michal.gala@student.upjs.sk

**Keywords:** nanoparticles, ecotoxicology, transcriptomics, *Arabidopsis thaliana*, biotic stress, abiotic stress

## Abstract

Transcriptomics studies are available to evaluate the potential toxicity of nanomaterials in plants, and many highlight their effect on stress-responsive genes. However, a comparative analysis of overall expression changes suggests a low impact on the transcriptome. Environmental challenges like pathogens, saline, or drought stress induce stronger transcriptional responses than nanoparticles. Clearly, plants did not have the chance to evolve specific gene regulation in response to novel nanomaterials; but they use common regulatory circuits with other stress responses. A shared effect with abiotic stress is the inhibition of genes for root development and pathogen response. Other works are reviewed here, which also converge on these results.

## 1. Introduction

Recent advances in nanoscience have expanded the range of applications for novel nanomaterials and driven their production to the industrial scale. In parallel, their emissions to the environment have increased up to the limits where environmental impact needs to be evaluated. Global estimations indicate that landfills and soils receive the largest share of the production volumes, followed by emissions into the aquatic environment and air [1,2]. Fate and transport studies further suggest that disposed nanomaterials end up in natural habitats at concentrations that might pose a risk for living organisms.

**Figure 1:**
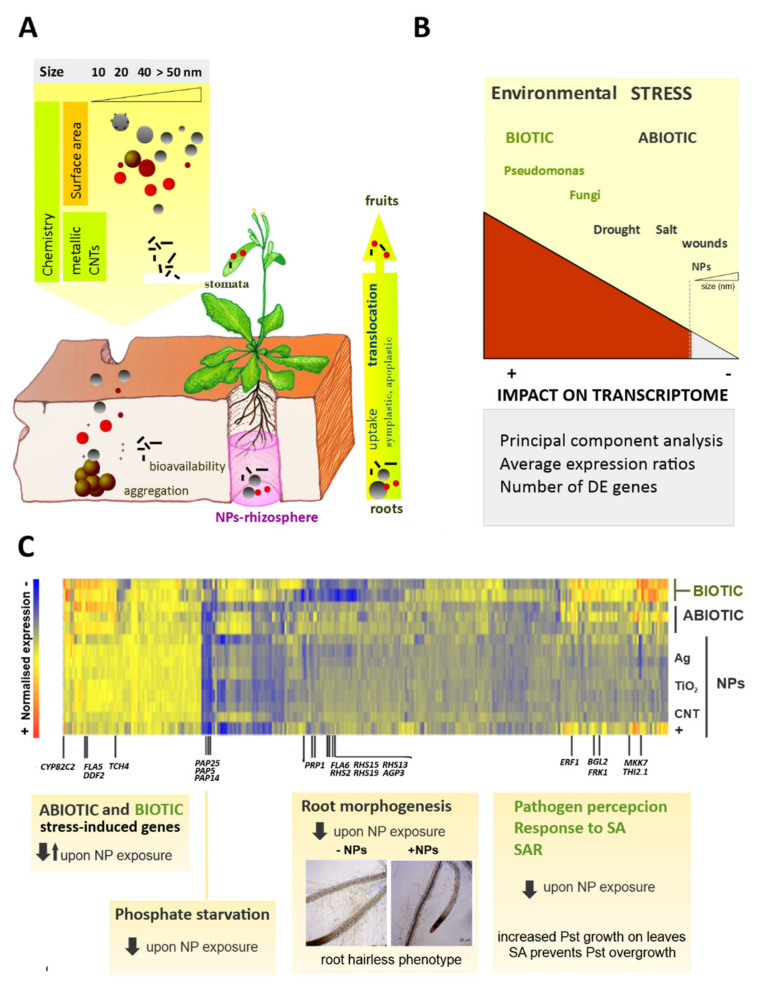
Transcriptomics approaches to evaluate nanoimpact in planta (**A**) Nanoparticles (NPs) released to the environment can enter into the biosphere and interact with plants, the primary producers in the food chain. NPs uptake occurs through the plant roots. These potentially toxic compounds can be translocated from the roots to the leaves and eventually reach the fruits. (**B**) The impact of NP exposure on plant transcriptome can be compared with other environmental challenges. Arabidopsis microarrays provided expression data for a large number of plant genes under different biotic or abiotic stress conditions, including early exposure to NPs. Unsupervised techniques like principal component analysis (PCA) summarize gene expression data and show that biotic stress is the main source of variation, whereas exposure to NPs causes little changes in the transcriptome. Comparative analyses of average expression ratios and the number of differentially expressed (DE) genes also support the low impact of NPs on the plant transcriptome. (**C**) DE genes were classified according to gene ontology (GO) categories, and enrichment was studied. The heat map shows the under-expression of genes involved in phosphate starvation, root morphogenesis, defense response to pathogens, and salicylic acid (SA) signaling. The expression of several genes that modulate systemic acquired resistance (SAR) is decreased upon NP treatment. Related phenotypes under NP exposure include a reduction in the number and length of root hairs and increased survival of *Pseudomonas aeruginosa* (Pst) in plant distal leaves.

Since plants represent by far the largest interface between the environment and the biosphere, they will be the first barrier for nanoimpact. Consequently, there is a need to evaluate the toxicological effects of nanomaterials in photosynthetic species as a way to assess for their ecological impact [3,4,5]. Together with standard toxicological methods, omics technologies are also available to quantify nanoimpact in plants. This review is focused on transcriptomics efforts carried out to approach the phenomenon of nanoimpact in plants and the global conclusions that can be drawn from these studies (Figure 1).

## 2. Physico-Chemical Properties of Nanomaterials and Implications for Plant Toxicology

Nanomaterials are defined as materials with at least one dimension in the nanoscale (1–100 nm). If all their external dimensions do not differ significantly within the nanoscale, the term nanoparticle (NP) is preferred to distinguish these objects from others like nanofibers or nanoplates. At the nanoscale, materials behave very differently compared to larger scales, and they often display unique chemical and physical properties. This makes the nanotechnological transformation of matter a promising field to develop new products and processes. On the counterpart, their unique properties allow novel materials to interact unexpectedly with biological systems [6].

The main distinction can be established between two different kinds of NPs: (1) naturally occurring, which are mostly amorphous and chemically and physically highly variable particles of nanometer dimensions, and (2) custom man-made artificial nanoparticles with highly reproducible physico-chemical properties. Natural NPs are usually generated in an uncontrolled way by natural processes (i.e., volcanic activity), or they are undesirable by-products of human activities processes. On the other hand, synthetic NPs are engineered materials with well-defined dimensions and controlled physical and chemical properties, which are the major factors driving their industrial applications.

Among the fundamental physical properties of NPs that have to be considered are overall size, surface area, and mechanical properties [6]. The overall size of NPs impacts the mechanism by which NPs can spread through the plant vascular system. Uptake of NPs by plant roots and translocation to upper tissues has been demonstrated for different species when fed with a suspension of NPs in synthetic media. Both apoplastic and symplastic transport of NPs may also occur under natural growth conditions, with a size limit for symplastic transport in most plants [4,7,8]. NPs with a diameter below 5 nm can translocate through the pores of the cell wall, 8–20 nm particles can move preferably between cells through plasmodesmata, and larger >50 nm particles can be internalized by the endocytosis [3]. Other factors affecting the amplitude of nanotoxicity for plants are the shape and surface area of the NP, which have been studied using crop plant models [9].

Regarding chemical properties, a large variety of chemical compositions and surface coating exist [6]. Metallic NPs are prepared from silver (Ag), gold (Au), copper (Cu), or metal oxides like TiO_2_. Carbonaceous nanofibers include single-well and multi-well carbon nanotubes (CNTs). Semiconductors such as silicon and ceramic are also used to make NPs. Polymeric NPs, on the other hand, are mostly colloid solids produced from polycaprolactone, polyacrylate, alginate, etc. The chemical composition of NPs, as well as their coating, can further regulate the chemical stability (e.g., changes in the redox state), overall reactivity, covalent attachment, and persistent binding to biomolecules. In most toxicological studies, metallic NPs exhibit the highest toxicity for living organisms, independently of taxa, and this has been strongly connected to the release of metal ions that induce the formation of reactive oxygen species (ROS) [10,11,12]. On the other side, carbonaceous and organic NPs show very low toxicity, or moreover, they might exert positive effects on plant growth or resistance to other environmental stress conditions. Nanomaterials can be made from different chemical compositions, in order to deliver faintly available nutrients and growth-promoting compounds, exhibiting beneficial rather than toxicological effects for plants [13,14,15,16].

Aside from the physico-chemical properties of individualized NPs, the formation of NP clusters and their aggregation induced by the medium or the environment can further impact the overall nanotoxicology. An aggregation process can dramatically increase the overall size of NP clusters and show a varying biological behavior of organisms. Overall, NP aggregates have been shown to induce decreased toxicity compared to individualized NPs in plants [17,18]. NP in the environment undergoes aging processes such as chemical transformation, aggregation, and disaggregation. The interplay between these processes and the NP transport determines the fate and ultimately the phyto-toxicological potential of NPs (Figure 1A).

## 3. Applications, Production, and Release to the Environment

Engineered nanomaterials represent a fast-growing market, as they have a high potential for product adoption in a variety of industries including aerospace and vehicle production, construction, chemical catalysis, or agri-food sectors [15,16,19]. Rapid developments in biomedical technology and pharmaceutical research are also expected to augment the industrial production of NPs in the years to come [20,21]. These materials are increasing their presence in consumer products and domestic devices, mostly as coatings to improve mechanical, optical, or antibacterial properties. As examples, NPs are now used in the manufacture of crack-resistant paints, scratchproof eyeglasses, transparent sunscreens, ceramic coatings for solar cells, or self-cleaning fabrics. The use of nano-scaled particles instead of their counterpart bulk materials provides advantages and increases the competitiveness of several market products. Nanoparticles of titanium oxide used in sunscreens, for example, have the same chemical composition as the larger white titanium oxide particles used in conventional products for decades, but nanoscale titanium oxide is transparent. Silver nanoparticles continuously discharge Ag^+^ ions, and they are used in clothing to kill the bacteria known to cause undesirable odors. Silica (SiO_2_) is a part of the normal mix in conventional concrete, but the use of its nano-form improves particle packing and mechanical properties of the concrete. Cerium oxide (CeO_2_) nanoparticles can switch from oxidation to reduction catalysts, and they have emerged as fascinating and lucrative material in biological fields such as biomedicine, drug delivery, and bio scaffolding [22].

As a result of the increasing demand by the market, the production and consequent release of NPs to the environment is growing more and more every year. For very frequently produced NPs like CeO_2_-, SiO_2_- and Ag-NPs, recent surveys estimated the annual global production volumes over 100,000, 1000–10,000, and 100–1000 t/a, respectively [20]. Probabilistic modeling has been used to predict the flow of released NPs to environmental compartments in the next years and to quantify their amounts in different environmental sinks [20,23]. Although for most environmental compartments NPs pose a relatively low risk of toxicity, organisms residing near NP “point sources”, like production plant outfalls and waste treatment plants, may be at increased risk. The model also indicates that the concentrations of NPs in soil and sediments will be higher than those in water or air. In agreement with existing measurements, modeling of NP dispersion, transport and fate predict that soils will be the final sink and major contaminant source of NPs released into the environment. Taking into account NPs life cycle, as well as the differences in their transport and stability, predicted concentrations of NPs in agricultural soils yield up to 10 μg/kg for 2050 [20]. For sludge-treated soil areas, for example, predicted concentrations might be as much as 40-fold higher. 

The investigations of NP production volumes, release, and persistence into different environmental compartments, as well as their toxicological effects, increased the perception of the risk that novel nanomaterials represent for environmental and human health [24,25]. As a consequence, more research was prompted to define ecotoxicological limits of exposure to NPs.

## 4. The Scales of Ecotoxicity for Nanomaterials Using Plant Assays

The investigation of potential risks for living organisms has resulted in the development of an ecotoxicological scale for nanomaterials. NPs are included in the list of chemical substances for which environmental effects are monitored and regulated by different countries. By using green algae and plant assays, nanomaterials have been classified in both aquatic and terrestrial ecotoxicity categories, where they rank from harmful to very highly toxic [26]. In aquatic environments, Ag, Au and Fe NPs result in being very highly toxic with EC_50_ values of <0.1 mg/L, followed by other metallic (Zn, Cu) NPs with EC_50_ = 0.1–1 mg/L (highly toxic), Cd and Ti NPs (moderately toxic, EC_50_ = 1–10 mg/L) and Graphene/Carbon NTs (slightly toxic, EC_50_ > 10–100 mg/L). In terrestrial ecosystems, only Ag NPs are classified as toxic in plant assays, having effects at EC_50_ > 10–100 mg/kg dry-weight soil. 

This classification only takes into account the chemical composition of NPs, without discrimination among sizes in the nanoscale or surface properties. As expressed before, these properties are important factors that govern NP stability and mobility as a colloidal suspension, and likely they will influence interactions with algae in natural aquatic systems or with rhizosphere and plant roots in terrestrial environments [27]. Thus, the phytotoxicity of nanomaterials in natural environments could largely differ from what is expected from standard ecotoxicological assays.

## 5. Presence and Generational Transmission of Nanomaterials in Crop Plants

An early study that stimulated further research about the genetic effects and potential toxicity of nanomaterials for plants was performed by Lin et al. [28]. These researchers used transmission electron microscopy (TEM) to demonstrate the uptake and transportation of NPs in crop plants. Rice plants were seeded in a suspension of natural organic matter to mimic freshwater ecosystems, and different concentrations of CNTs were added to the suspension. Seeds were kept in this germination medium for two weeks until they were transplanted to soil pots without further NP treatment. Tissues of plants at various developmental stages were sampled for TEM monitoring to evidence the translocation of CNTs from roots to stem, and from stem to leaves. However, more interestingly, the study showed that nanomaterials that were accumulated in the first generation of exposed plants could be transmitted to the second generation through seeds. Since rice provides food crops of over half the world’s population, this work suggested the potential impact of disposed nanomaterials on the food chain and raised important concerns about the genetic consequences of plant–NPs interactions. Subsequently, efforts to investigate these consequences using genome-wide technologies were pushed forward in other crop plants like tomato [29] or the model species *Arabidopsis thaliana*.

## 6. Transcriptomics Studies in Plants to Evaluate Nanoimpact

A powerful approach to determine how an organism responds to a particular environmental challenge is to determine how it changes the expression of its genome [30]. In this line, transcriptome studies to approach nanoimpact have been performed in several photosynthetic species, from unicellular green algae [31] to higher vascular plants like tomato [29], rice [32], or *A. thaliana* [33,34,35,36,37,38,39,40,41]. Thus, transcriptional data covering a range of species for which genomics tools are fully developed are available to assess for nanoimpact on plants. Omics technologies have the power to shift the research on plant-NP interactions from low-throughput, single end-point bioassays to high-throughput discovery [42].

While information on the transcriptional effects of NP exposure is available, the findings are somewhat contradictory. Several studies show a strong effect on the transcription of stress-related genes and suggest high toxicity for the plant, whereas some others do not find significant transcriptional changes and have concluded that NPs are unlikely to produce any adverse effect for the plant. In parallel, physiological and biochemical changes observed upon NP exposure include both significant reduction and significant promotion of plant growth, elongation or shortening of plant roots, the formation of ROS, or no indications at all for oxidative stress. One reason for these apparently contradictory conclusions is that many of these studies are focused on specific effects for a given, chemically defined type of NP, and other factors like size-dependent effects are not taken into account. Moreover, a very small fraction of plant genes is used as markers for conventional toxicological studies, and they are mostly included in predefined functional categories, such as oxidative stress response, which are not very informative at the morphological level. As a consequence, there is a missing node to relativize the toxic effects of NPs.

### 6.1. Green Algae

#### 6.1.1. Chlamydomonas Reinhardtii

Unicellular, green algae have been widely used as sentinel species for ecotoxicological studies both in freshwater and soil ecosystems [43]. Thus, one of the first attempts to establish the potential impact of novel nanopollutans by using transcriptomics approaches was carried out in the model species C. reinhardtii [31]. Previous toxicological studies in this alga [44] evidenced that several types of NPs are able to penetrate the cell wall and induce the production of ROS, or cause cell damage by reacting directly with the biological membrane. The researchers used mRNA sequencing to evaluate the effects of exposure to four different (nZnO, nAg, nTiO_2_, and CdTe/CdS quantum dots) metal-based NPs, with diameters ranging between 20 nm for nZnO and 1–10 nm for the other three types of NPs. Transcript fold change between two conditions, NP exposure vs. no exposure, was used to determine differential expression; and genes were considered as differentially expressed (DE) if they met a ≥2 -fold change ratio between both conditions. The researchers found that NP exposure resulted in largely different transcriptomic responses. Surprisingly, only the exposure to nZnO induced the transcription of GSTS1, HSP22C, and HSP70A, genes considered as the main markers for oxidative stress, since they encode for a glutathione-S transferase and heat-shock proteins induced by excess H_2_O_2_ or singlet oxygen. The effect of the other three types of NPs was actually a down-regulation of stress-related genes. The study also revealed that TiO_2_ and ZnO NPs and CdTe/CdS quantum dots impact the proteasome machinery and produce proteasome inhibition. Interestingly, a consistent effect of all types of tested NPs was the transcriptional inhibition of photosynthesis-related genes, suggesting toxicological effects in the chloroplast. In this line, biochemical studies in the planktonic species Scenedesmus obliquus [45] show that photocatalytic activity of nTiO_2_ can damage algae by directly reacting with chloroplast photosynthetic machinery and generating ROS. The effect on other stress-related genes that was observed in transcriptional studies has been interpreted as a stimulation of the plant defense system in order to scavenge produced ROS.

An important concern about the toxicological effects reported for nanoparticles is that many of them are associated with supra-environmental exposure concentrations [46]. Most studies in green algae have been conducted through acute toxicity testing (short-time exposure to high doses), even though environmental effects are likely to be better assessed by chronic toxicity testing (long-time exposure to low doses). Thus, the impact of NPs on freshwater ecosystems at environmentally relevant concentrations is not clear from these experiments, and many questions about how surrounding biota can modify the toxicology of nanopollutants for photosynthetic organisms remain to been elucidated.

#### 6.1.2. Microcosm Transcriptional Response

An interesting approach to determine nanotoxicity for freshwater ecosystems under more realistic environmental conditions was performed by Lu et al. [47]. In a microcosm experiment including aquatic eukaryotic algae, fungi, zooplankton, and bacteria (i.e., heterotrophic bacteria and cyanobacteria), a meta-transcriptomic analysis was used to decipher the toxic effects of Ag NPs (10 nm), at relatively low doses (10 μg/L), and upon long-term (7-days) exposure. It was found that photosynthetic eukaryotes were much more tolerant to Ag NPs than cyanobacteria and displayed a number of potential Ag NPs detoxification mechanisms, which involved increasing nitrogen and sulfur metabolism, over-expression of genes related to translation and amino acids biosynthesis, and the promotion of bacterial-eukaryotic algae interactions. Thus, transcriptomic analysis reveals that photosynthetic organisms overcome exposure to nano-pollutants by triggering a set of complex responses above the transcriptional activation of genes involved in ROS detoxification.

### 6.2. Higher Plants

In model plant species, genome expression microarrays are available to profile transcriptome under different environmental challenges. Most genome databases for model organisms link gene annotation to microarray expression platforms. Microarrays provide a standard mean for normalization of gene expression data which facilitates comparisons among multiple conditions, even when data are originated from different experiments. With this regard, several efforts were put into the transcriptional characterization of nanoimpact using genome-covering microarrays for higher plants.

#### 6.2.1. Tomato

Khodakovskaya et al. [29] integrated imaging and genetic technologies to approach the phenomenon of nanoimpact in tomato plants. By using photothermal and photoacoustic cytometry, they first mapped CNTs in roots, leaves, and fruits of plants fed with a suspension of these NPs. Next, they profiled transcriptional changes induced by CNTs by using a preliminary microarray covering approximately 1/4 of the tomato genome.

In their experiments, total mRNA was isolated from leaves and root tips of 10-day-old tomato seedlings growing on Murashige and Skoog (MS) medium, MS medium supplemented with CNTs (50, 100, 200 mg/L), or with activated carbon as a control for the effect of bulk, non-particulated material. Using a tomato microarray containing probes to interrogate over 9200 plant transcripts, the authors identified 91 and 49 transcripts in leaves and roots, respectively, that showed significant differences between the CNT-exposed seedlings and two controls. 

Within this set of DE transcripts, the researchers observed that several up-regulated genes in response to CNTs (i.e., genes encoding for several endoproteases, the heat shock protein HSF70, or the LeAqp2 gene encoding for a water-channel protein) can also be activated in response to specific biotic stress factors. This observation suggested that plants can sense the penetration of nanomaterials as a stress factor, similar to pathogens or herbivore attacks. Therefore, the authors introduced the idea that important stress-signaling pathways could be activated in response to the uptake of NPs, and accordingly, nanomaterials could have a significant impact on most of the major physiological processes in planta. Conversely, the number of DE genes extracted by statistical analysis represents a small fraction of the tomato genes covered by the microarray. More challenging are the physiological effects observed after feeding tomato plants with CNTs, including a significant increase in plant biomass production, while exposure to environmental stress in crop plants usually results in poor biomass accumulation. 

Other works in tomato [13] reported the induction of pathogenesis-related (PR) genes and the enhancement of biotic stress responses after exposure to chitosan-NPs. Both chitosan and chitosan-NPs were able to inhibit the development of wilt caused by Fusarium andiyazi in tomato plants and to elicit transcriptional responses characteristic of the induced systemic resistance, although these effects were not specific to the exposure to nanosized chitosan.

#### 6.2.2. Arabidopsis

The extensive genome annotation available for the model species A. thaliana and the consequent development of advanced post-genomics tools enabled a finer evaluation of nanoimpact in higher plants. Whole-genome expression microarrays were used initially to characterize gene expression changes in response to different nanomaterials, including ZnO nanopowder, a roughly characterized mixture of TiO_2_ NPs, and fullerene soot [39]. 

Plants growing in MS medium were exposed for seven days to a final concentration of 100 mg/L of NPs, and roots were harvested for microarrays analysis of gene expression. nZnO caused the most dramatic transcriptomic changes of the three nanomaterials under study, both in terms of the number of affected genes and in the magnitude of the impacts on gene expression. In addition, more genes were repressed than activated, suggesting that nZnO represented a severe stress condition for the plants. However, these experiments assayed the effects of a high concentration of NPs into a defined growth medium, and the authors themselves stated that an assessment of true environmental risk should be focused on more environmentally relevant NP concentrations. Nevertheless, the study showed that genes induced by nZnO and fullerenes include mainly ontology groups annotated as stress-responsive, including both abiotic and biotic stimuli. The down-regulated genes under nZnO exposure were involved in cell organization and biogenesis, whereas fullerenes largely repressed genes involved in electron transport and energy pathways. The counterpart effects of non-particulate, bulk materials on genome expression were not tested in this study, and thus, many of the transcriptional changes described by the authors could be non-specific of NP exposure.

##### Impact of Silver NPs Exposure on Arabidopsis Transcriptome

Silver NPs are the most widely used nanomaterials that enter into the wastewater and potentially damage the environment. Due to their antimicrobial properties, they are used in a wide variety of processes, including disinfection of domestic water or the production of antimicrobial coatings for textiles, house appliances, or biomedical devices. Many of these products contain silver nanoparticles that continuously release a low level of silver ions to protect against bacteria. Concordantly, the ecotoxicology of Ag NPs is complex because it may be related simultaneously to silver-specific and nanoparticle-specific biological effects. 

In a pioneer work by Kaveh et al. [33], transcriptome analysis offered a powerful tool to approach the complexity of plant responses to Ag nanopollutants. As the first step in this study, the potential toxicity of Ag^+^ or Ag NPs (20 nm) was evaluated for Arabidopsis plantlets grown during ten days in MS medium containing increasing concentrations of both factors, ranging from 0 to 20 mg/L. At low levels (1.0 and 2.5 mg/L), exposure to Ag NPs resulted in a significant increase in biomass with respect to untreated plants and respect to plants treated with Ag^+^, although exposure to higher concentrations resulted in a decrease in biomass for both treatments. A concentration of 5 mg/L, which resulted in moderate reductions in plant biomass, was chosen for microarray experiments. 

Further analysis of microarray data was based on expression fold-change values with respect to non-exposed plants. Exposure to Ag NPs resulted in differential expression of 375 genes, with a significant overlap with DE genes that responded to Ag^+^ treatment. The overlap suggested that Ag NP-induced stress originates partly from silver toxicity and partly from nanoparticle-specific effects. Many genes responding to both treatments were found to be involved in plant response to various stresses: up-regulated genes were associated with the response to metals and oxidative stress (i.e., genes encoding for vacuolar cation/proton exchanger, superoxide dismutases, cytochrome P450-dependent oxidases, and peroxidases), while down-regulated genes were more associated with response to pathogens, including systemic acquired resistance (SAR) against fungi and bacteria, and hormonal stimuli (auxin or ethylene signaling pathways). Interestingly, most overlapping genes, affected both by Ag^+^ and Ag NPs, were down-regulated.

On the other hand, a number of genes were found as differentially expressed in response to Ag NPs only. This set of genes more likely reflected the molecular mechanisms involved in NP-specific responses. The most up-regulated genes in this set were involved in salt stress response, which established a connection with the previous work in Arabidopsis, where genes related to saline stress were found to be induced upon exposure to both nZnO and fullerenes [39]. The set also included up-regulated genes encoding for a miraculin-like protein involved in the plant response to wounding, a myrosinase-binding protein induced during defense against insects and pathogens, and more intriguingly, a cluster of genes belonging to the thalianol biosynthetic pathway. In plants, these rare clusters are believed to be involved in the biosynthesis of stress-induced secondary metabolites that are required for survival under specific conditions, such as the exploitation of new environments.

Thus, transcriptome analysis of Arabidopsis plants exposed to silver provided some clues for a better understanding of the molecular mechanism of plant response to NPs and established a connection with physiological responses involved in both abiotic and abiotic stress sensing.

## 7. Comparative Analysis of Transcriptomic Changes Induced by Nanoparticles and Other Environmental Challenges

The studies described so far in this review did not introduce transcriptomic data from other types of environmental challenges to establish a comparison with NP exposure. Transcript fold change between 2 conditions, “NP-exposed” versus “non-exposed”, was used to determine differential expression, and genes were considered as DE if they met an arbitrary ≥2 -fold change ratio. However, without a comparison with other environmental stresses that cause significant changes in plant physiology and, conversely, in-plant transcriptome, fold-change ratios remain a very relative way to assess the toxicity of NPs. Thus, more experimental conditions able to mimic environmental sources of stress should be introduced in transcriptomic experiments, to normalize expression data and make them a powerful tool to assess for biological impact of NP exposure.

### 7.1. In Silico Comparison of Ag NPs with Abiotic Stressors

In silico analysis of microarray data from publicly available repositories (GEO and Array Express) allowed comparisons of transcriptomic changes induced by Ag NPs as described by Kaveh et al. [33] and four different types of abiotic stress induced by cold, heat, drought, or hypersaline conditions [48]. Although microarrays data used for the comparison were originated under different experimental conditions of plant growth, the study shed some light on the impact that NP exposure raises on the plant transcriptome. The comparison was based on the number of genes affected (DE genes) by each type of stress and showed that Arabidopsis plants responded to Ag NPs by changing the expression of a low number of genes (2.10% of the genome), whereas cold and salt stresses induced DE of the largest numbers (23.84% and 15.08% respectively). Comparison with Ag^+^ exposure showed that only a very low fraction of plant genes (111 genes) was responding specifically to the NP-form of Ag. Overview of metabolic/regulatory pathways revealed that ROS-associated genes were up-regulated by both Ag NPs and Ag^+^, in agreement with previous results. The analysis of enrichment in gene ontology terms showed that the set of 111 NP-responsive genes was enriched in three biological functions: response to fungal infection, anion transport, and cell wall/plasma membrane. Despite the shared similarity to Ag^+^, cold, and salt stresses, Ag NPs were proposed as a novel abiotic stressor to *A. thaliana*.

### 7.2. In Vivo Comparisons with Biotic and Abiotic Stressors

Since the range of the transcriptional response to stress depends on the growth conditions and developmental stage of the plant, the effect of different types of stressors should be evaluated in plants grown under similar conditions. To introduce comparable transcriptome data that provided in vivo rather than in silico evidence supporting the role of NPs as plant stressors, we [34] produced a set of 16 comparable transcriptome profiles to monitor early changes in gene expression upon NP and stress exposure. Synchronized plants were exposed to biotic stress by inoculation with the fungal necrotroph *Alternaria brassicicola* or the hemibiotrophic bacterium *Pseudomonas syringae*, or to abiotic stress by hypersaline conditions, drought, or wounding. We assayed NPs made of 3 different materials, Ag, TiO_2,_ and carbonaceous CNTs, together with their corresponding bulk components, and at different sizes in the nanoscale ranging from 10 to 80 nm for Ag NPs, and from 10 to 40 nm for TiO_2_ NPs. Since abscisic acid (ABA) hormone is a key regulator of abiotic stress responses, exogenous addition of ABA was assayed to test for remediating effects on CTN-exposed plants. This made a total of 9 different conditions where plants were exposed to NPs for 48 h before RNA was collected for transcriptome analysis.

**Figure 2:**
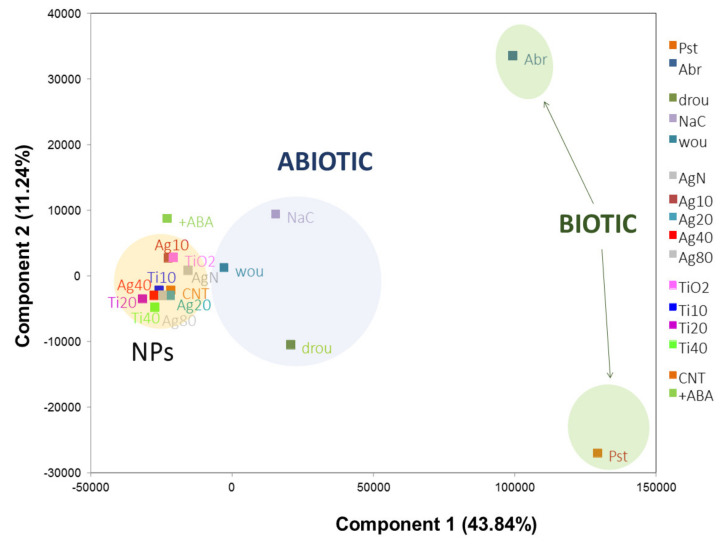
Principal Component Analysis of Arabidopsis microarray data performed by García et al. [34]. The first and second components of the analysis, which captured respectively 43.84% and 11.24% of the variation in data, were plotted to visualize similarities among samples. Plants were exposed to biotic stress by inoculation with the necrotrophic fungus *Alternaria brassicicola* (**Abr**) or the hemibiotrophic bacterium *Pseudomonas syringae* (**Pst**), and to abiotic stress by hypersaline conditions (**NaC**), drought (**drou**), or wounding (**wou**). For NP exposure, plants were grown in MS medium containing NPs made of 3 different materials -Ag, TiO_2,_ or carbonaceous nanotubes- and at different sizes in the nanoscale. **Ag20**, **Ag40**, **Ag80**, and **AgN** represent the addition of Ag NPs of 10-, 20-, 40- and 80-nm diameter, or AgNO_3_ bulk material, respectively. **Ti10**, **Ti20**, **Ti40**, and **TiO2** indicate the addition of TiO_2_ NPs of 10-, 20-, 40-nm diameter, or TiO_2_ bulk material. **CNT** indicates the addition of carbon nanotubes. Since Abscisic Acid (ABA) hormone is a key regulator of abiotic stress responses, exogenous addition of ABA (**+ABA**) was assayed to test for remediating effects on CTN-exposed plants. Transcriptomic data are available at www.ebi.ac.uk/arrayexpress under the accession number E-MTAB-3331.

To get an overall picture of nanoimpact compared to other stresses, the changes that occurred in the transcription of 26,184 Arabidopsis genes were examined using Principal Component Analysis (PCA, Figure 2). Genes that were DE under NP-treatments were extracted by statistical analysis to define a set of 351 NP-responsive genes, irrespectively of material or size of the NP. A more detailed study of expression patterns characteristic of NP exposure was performed using GO enrichment analysis and clustering methods (Figure 1). The main findings of transcriptomic analysis reported in this work are discussed next and referred to other studies that converged on these results.

## 8. Transcriptomic Signatures of NP Exposure in Plants

### 8.1. NP Exposure Produces Specific Transcriptional Patterns Related to Abiotic Stress

Analysis by PCA was able to detect major trends in the transcriptome data under different biotic or abiotic stress conditions, including NP-treatment. As shown by the scatter plot (Figure 2), transcriptomes of plants exposed to different types of NPs, irrespectively of material or size in the nanoscale, behave similarly and cluster together. Abiotic stress conditions by wounding, followed by hypersalinity and drought, are the closest conditions to NP exposure. On the other extreme of the plot are biotic stresses by a necrotrophic fungus or by a hemi-biotrophic bacterium. In agreement with the conclusions reported in previous works [33,39,48], this analysis was able to show that exposure to NPs of different compositions or sizes in the nanoscale induces common transcriptome signatures in plants.

### 8.2. NP Exposure Has a Low Impact on the Plant Transcriptome

**Figure 3:**
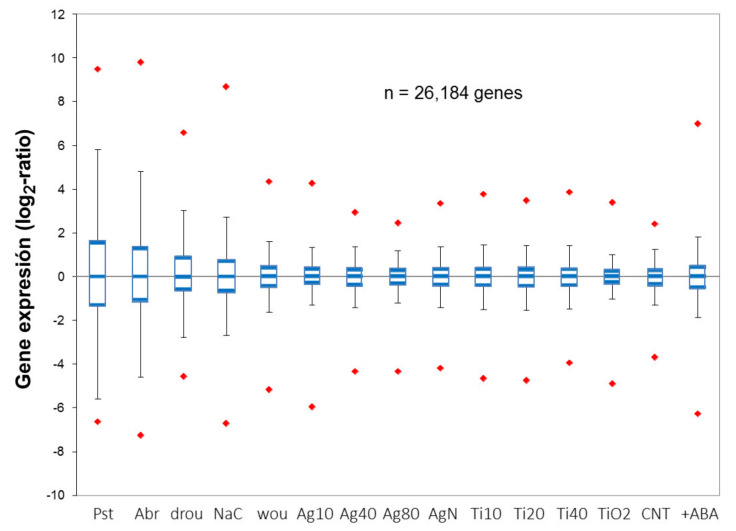
Box-plot distribution of the expression ratios from 26,184 genes represented in AtV4 microarrays [34]. Boxes represent 10th and 90th percentiles, with the median (Q2) in the middle line of the box. Minimum and maximum values are shown by the end of the vertical lines (whiskers) and upper or lower outliers by red diamonds. **Pst** and **Abr** denote biotic stress by bacteria or fungi, respectively. **drou**, **NaC**, and wou abiotic stress by drought, salt, and wounding. **Ag20**, **Ag40**, **Ag80**, and **AgN** represent the addition of Ag-NPs of 10-, 20-, 40- and 80-nm diameter, or AgNO_3_ bulk material, respectively. **Ti10**, **Ti20**, **Ti40**, and **TiO2** indicate the addition of TiO_2_-NPs of 10-, 20-, 40-nm diameter, or TiO_2_. **CNT** and **+ABA** indicate the addition of CNTs or CNTs plus ABA.

Both PCA analysis and box-plot representation of normalized expression ratios for all the genes in the microarray show that transcriptomic changes induced by NPs are in the lowest order of magnitude when compared to other sources of stress (Figure 3).

The transcriptomic response, as measured by the distribution of expression ratios across different stress conditions, increases gradually from NPs to wounding, saline, and drought stress. On the other extreme, the most dramatic transcriptomic changes that could be observed in comparable experiments corresponded to biotic stress induced by a microbial pathogen, Pst, with a life cycle very adapted to the plant host. The low impact that NPs cause on transcriptome is also reflected in the number of DE genes that respond to different stresses, as was previously stipulated [33,48]. Transcriptome characterization by mRNA sequencing to investigate the toxic effects of aluminum NPs [37] also showed that a discrete number of genes are affected by the nanoparticulated form of this metal. Considering data from different studies with metallic NPs, bulk materials have a greater impact on gene expression than their corresponding nanoparticulated forms. 

#### Size-Dependent Effects of NPs

Although NPs of different sizes cluster together in the first two components of PCA, a consistent trend is observed in the expression ratios; the trend points to a higher impact of the smallest sizes in the nanoscale. This might be reflecting the fact that smaller nanoparticles can be translocated more efficiently to the different tissues in the plant, where they exert more physiological and transcriptional effects than restricted to roots.

### 8.3. Transcriptional Inhibition as a Feature of Early NP-Exposure

**Figure 4:**
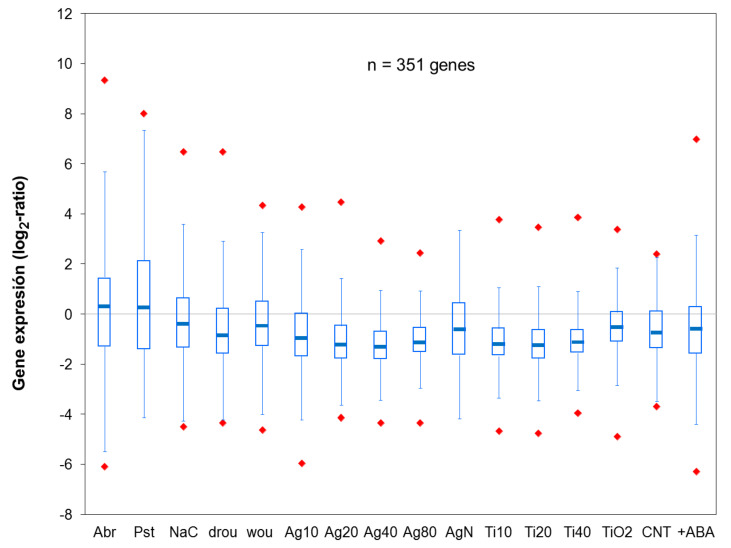
Box-plot representation of expression ratios for 351 NP-responsive genes [34]. Quartile distribution of the expression ratios for the gene set is shown, where boxes represent Q1 and Q3 quartiles and the middle line of the box is the median (Q2), while whiskers show the minimum and maximum values and red diamonds represent outliers. (**Pst**, **Abr**) biotic stresses by bacteria or fungi, (**drou**, **NaC**, **wou**) abiotic stresses by drought, salt or wounding, (**Ag20**, **Ag40**, **Ag80**, **AgN**) addition of Ag NPs (10-, 20-, 40- and 80-nm diameter) or AgNO_3_, (**Ti10**, **Ti20**, **Ti40**, **TiO2**) addition of TiO_2_ NPs (10-, 20-, 40-nm diameter) or bulk TiO_2_, (**CNT**, **+ABA**) addition of CNTs or CNTs plus ABA. Notice that most genes are repressed under NP treatments.

The significant analysis of microarrays (SAM) was used to identify a set of 351 genes that were differentially expressed in response to all types of NPs (Figure 4).

Box-plot representation shows that median values of expression for this set of NP-responsive genes are under the 0 baseline for all the conditions where NPs were tested. This is also true for the Q1 quartile, except in the plants exposed to CNTs but supplemented with ABA. Overall, down-regulation considering the whole genome dataset can also be concluded under all conditions where NPs were added to the plants. This confirms that transcriptional repression is one of the main effects of NP exposure in Arabidopsis, as it was observed in the study with nZn [39] where more genes were found to be repressed than activated. Other transcriptomic approaches in different species, like mRNA sequencing in *C. reihardii* [31], also suggested the down-regulation of gene expression as a transcriptomic feature common to different NPs exposure. Studies in upper plants where exposure to NPs was conducted over longer periods (i.e., weeks) do not clearly show transcriptional repression. Since repression on a large number of genes cannot be maintained for a long time without compromising plant survival, this likely reflects the ability of plants to adapt signal sensing and transcriptional machinery to large periods of environmental stress.

### 8.4. NPs Repress Genes That Are Activated during Plant Response to Biotic Stress

Several works had previously pointed towards the association of transcriptional responses to NP and biotic stress. However, the way in which these connections work was not clear. Kaveh et al. [33] showed that up-regulated genes that were DE in response to both Ag^+^ and Ag were mostly involved in response to metals and oxidative stress, whereas down-regulated genes were more associated with ethylene signaling pathways and SAR response against fungi and bacteria. They suggested that the downregulation of genes involved in biotic stress response can be understood as a prioritization of plant defense mechanisms under Ag NP- and Ag^+^-induced stress.

**Figure 5:**
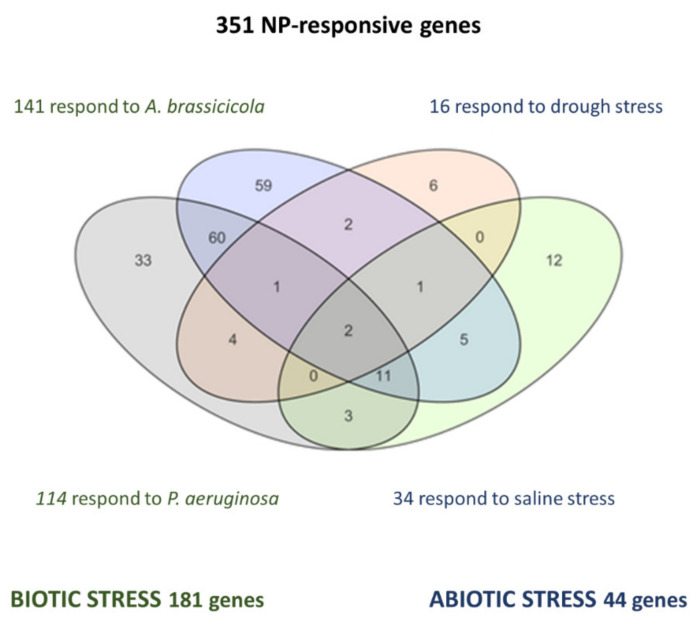
Subsets of biotic or abiotic stress-responsive genes in the set of 351 DE genes [34].

Comparison of transcriptome data produced under exposure to NPs and different sources of biotic stress shows that a number of NP-responsive genes that are repressed upon exposure to NPs are activated under biotic stress (Figure 1C and Figure 4). This is more in clear when Venn diagrams are used to extract, from the set of 351 NP-responsive genes, subsets of genes that are also DE in response to biotic stress by *A. solanii* or *P. syringae* (Figure 5). Over-expression induced by the bacterium in the latter set increases median ratio values >2, whereas all NP treatments strongly decrease median expression ratio below 0 baseline.

Clustering analysis shows that key genes involved in recognizing pathogens and the transduction of signals to activate SAR are repressed under treatment with NPs (Figure 1C). The negative effect of NPs on biotic stress signaling was evidenced in experimental infections with *P. syringae*. Plants that were exposed to NPs before inoculation with bacteria were over-colonized by the pathogen. The addition of salicylic acid (SA) was able to prevent this effect in distal leaves, suggesting a role of NPs in the inhibition of biotic stress signaling [34].

**Figure 6:**
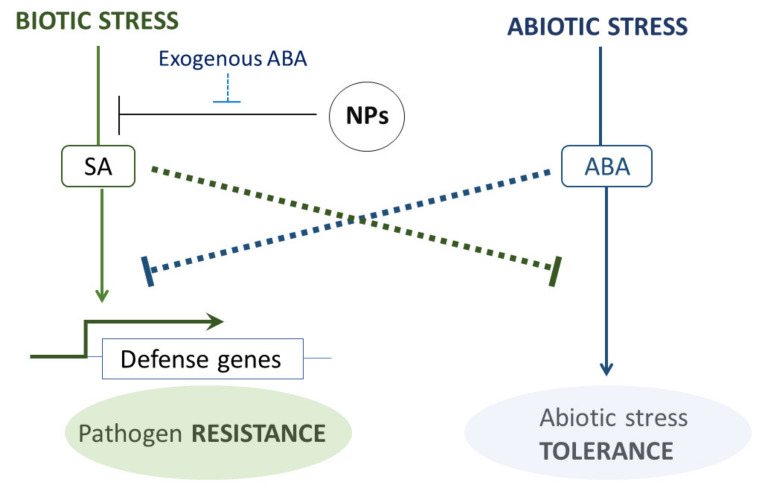
Early effects of NP exposure on stress-signaling pathways, as inferred from transcriptome analysis and phenotypes observed by García et al. [34]. NPs interfere with biotic stress pathways by transcriptional inhibition of pathogen-sensing and SAR-signaling genes. Exogenous addition of ABA can increase the expression of affected genes and remediate some transcriptomic effects upon NP exposure.

Regarding abiotic stress, the extraction of genes that are also DE in response to hypersaline conditions showed that up-regulated genes under saline stress are mostly down-regulated under NPs. However, up-regulated genes in response to drought stress are also up-regulated in NP-exposed plants. Interestingly, the addition of ABA to CNT-treated plants recuperates, in most cases, normal expression values for NP-responsive genes. ABA is a key hormone in the regulation of abiotic stress signals, which converge in the activation of callose deposition and other physiological responses to increase plant tolerance to adverse conditions. Crosstalk between abiotic and biotic signaling pathways ensures that there is not a redundant activation of these responses. As observed from our transcriptomics data, the effect of NPs on these pathways might be schematized (Figure 6).

### 8.5. Exposure to NPs Represses Root Morphogenesis and Phosphate-Starvation Responses

GO enrichment analysis foregrounded some functional categories in the genes affected by NP exposure. Genes involved in phosphate starvation responses and root epidermal cell differentiation were strongly repressed in plants treated with NPs (Figure 1C). Both categories are physiologically related because phosphate starvation induces important changes in root morphology by promoting the inhibition of primary root growth and the formation of lateral roots, as well as the proliferation of root hairs [49]. Microscopic examination revealed a “hair-less”-like phenotype in plants exposed to NPs, with a significant reduction in the density of hairs produced by the root tips. The “hair-less” phenotype was reverted by exogenous addition of salicylic acid, but not by other hormones like ABA or Jasmonates [34]. Stress signals shape the plant root and can induce the proliferation of root hairs to increase nutrient and water absorption or, conversely, reduce root hair formation to minimize the uptake of toxic substances or the interaction with soil-borne pathogens. 

Other works supported phenotypic and transcriptional effects of NPs on root inhibition [7,50]. Since roots are the main organ involved in the uptake of NPs by the plant, transcriptional inhibition of root morphogenesis and nutrient uptake would offer a physiological mechanism to prevent NP-induced toxicity.

## 9. Future Perspectives

Omics technologies allow a comprehensive understanding of biological responses to environmental challenges. Recently, considerable research has been carried out to characterize plant responses to novel nanomaterials by using transcriptomic approaches. Transcriptomic changes induced by NPs and most common abiotic and biotic stressors can be compared to provide mechanistic insights into plant–NPs interaction. Moreover, comparative transcriptomic analysis has proven to be mean to predict phenotypes associated with NP-exposure, i.e., the increased colonization of leaves by phytopathogens and the subsequent effect on SAR response. From these studies, transcriptional repression, the inhibition of biotic stress responses, and root-hair development can be headlined as early effects of NP exposure on plants (Figure 1).

Extensive data mining on transcriptomics results by different researchers is a pending issue that should shed more light on the plant responses to novel nanomaterials and the risk they pose. Although some studies suggest acute toxicity at the cellular level by the formation of ROS, this is unlikely to happen at environmentally relevant concentrations. On the other hand, higher plants are able to deploy different physiological responses that increase their tolerance to adverse conditions, and they made use of them during exposure to NPs. The low impact that NP exposure produces on the transcriptome, compared to other environmental stress conditions, suggests that plants might be used as a barrier for bioremediation against ecotoxic effects of nanoparticles. Moreover, metal phytomining and other biotechnological uses of nanomaterials could be adapted to plants with several advantages for other host species.

## Data Availability

Microarrays hybridization data can be found at www.ebi.ac.uk/arrayexpress under the accession number E-MTAB-3331.
